# Trophic relationship between *Salix* flowers, *Orthosia* moths and the western barbastelle

**DOI:** 10.1038/s41598-023-34561-6

**Published:** 2023-05-05

**Authors:** Grzegorz Apoznański, Andrew Carr, Magnus Gelang, Tomasz Kokurewicz, Alek Rachwald

**Affiliations:** 1 Department of Vertebrate Ecology and Paleontology, Institute of Environmental Biology, Wrocław University of Environmental and Life Sciences, Wrocław, Poland; 2 Forest Ecology Department, Forest Research Institute, Sękocin Stary, Raszyn, Poland; 3 Gothenburg Natural History Museum, Göteborg, Sweden; 4 Gothenburg Global Biodiversity Centre, University of Gothenburg, Göteborg, Sweden

**Keywords:** Ecology, Zoology

## Abstract

We present the results of a study which describes the relationship between the western barbastelle *Barbastella barbastellus* a highly specialised moth predator, and its prey—moths of the genus *Orthosia*, another selective animal known to converge around a dominant producer of pollen and nectar in early spring—willow trees *Salix* sp. In order to describe this trophic relationship, we conducted acoustic recordings at five paired sites (willow/control tree) in proximity to known barbastelle hibernation sites (Natura 2000: PLH080003 and PLH200014) beginning in mid-March 2022 after the first willow blossom sighting. Our study confirms a relationship between willow trees and barbastelles during early spring, as their activity around them was significantly higher than control sites. We also explore the activity of barbastelles over time, finding that activity levels around willows significantly decrease from the night of the first recorded bat, while the abundance of non-moth specialist bats remains consistent. Short-time importance (directly after hibernation) of willows for a moth specialist bat is probably due to other species blossom, attracting alternative prey, and in consequence—the bat. This newly described relationship should influence current conservation measures aimed at barbastelles.

## Introduction

The western barbastelle *Barbastella barbastellus* (Schreber 1774) is a species known to hibernate in relatively cold temperatures ranging from − 3° to 6.5°^1–3^. In well-isolated deep underground systems such as Nietoperek Natura 2000 site PLH080003 barbastelles tend to occupy colder parts, often near entry points^3–5^. Their preference for cold is further apparent in that they are frequently found in less isolated hibernacula such as bunkers, cellars^6,7^ and even trees^8^. This adaptation allows the species to be among the first bats in central Europe to finish hibernation, usually leaving wintering sites mid-march^5^. Early spring poses a severe challenge for temperate zone bats. After months of dependence on energy reserves gathered before hibernation, they urgently need to find energy sources (prey). Simultaneously, insect abundance in early spring is still limited (life cycle, winter dormancy maintained by photoperiod and temperature)^9–11^. While most European species depend on prolonged hibernation until prey becomes more numerous barbastelles are already active. This behavioural adaptation is interesting because barbastelles are highly specialized moth predators rather than opportunistic foragers^2,12,13^. Evolutionary armed barbastelles with sophisticated echolocation, utilizing low amplitude calls^14^, allows them to bypass the acoustic defence of owlet moths Noctuidae—an adaptation based on ultrasound hearing combined with evasive manoeuvres triggered by high-intensity echolocation calls^15,16^. This trait makes barbastelles an excellent aerial hawking animal whose diet consists of over 90% moths^12,13^. The only other European species: that is known to effectively hunt owlet moths are long-eared bats—*Plecotus* sp.^17,18^, which we also included in our analysis. In this case, *Plecotus* sp. uses passive hearing^17,19^.

To consider why a highly selective bat leaves hibernation so early when its prey availability is restricted, we explored the available data on barbastelle prey^12,13^. We discovered that in the early spring season, most of its prey consists of the genus *Orthosia*—a highly specialized genus of noctuid moths, which includes several large species, all of which, as adults, feed on the pollen and nectar of *Salix* flowers at night^20^. Willow trees and shrubs are mainly formed by the goat willow *Salix caprea* (Linné, 1753). Since this species forms numerous hybrids in Europe with other species of the genus *Salix*, we will use the name *Salix* sp. Dependent on willows—a primary producer of nectar and pollen in early spring^21,22^, *Orthosia* moths are active earlier in the season than most other owlet moths^23^. A few other nocturnal moths feed on *Salix* flowers early in the season, such as *Eupsilla, Conistra* and *Agrochola*, as well as some species of Geometridae and Pterophoridae, but the most prominent genus is *Orthosia*^20,24,25^. Several other insect taxa visit early blossoming trees, such as bees (*Hymenoptera,* Apidae) and hoverflies (*Diptera,* Syrphidae). However, none of those groups represents a viable food source for the moth specialist barbastelle.

We designed a study based on pairwise deployment of acoustic detectors near hibernation sites to investigate this potential phenomenon. We hypothesize that in early spring, barbastelle activity will be significantly higher around flowering willows when compared to sites with tree species that flower later in the season. Additionally, we predict the activity will be large at the beginning of the vegetative season and procedurally decrease as other foraging options develop.

The western barbastelle is one of Europe's most threatened tree-dwelling bats^26^. it is included in Annexes II and IV of the EU Habitats Directive and is classified as Near Threatened in Europe^27^. We designed an experiment to better understand this species' ecology in a crucial period of the year and to improve conservation measures.

## Materials and methods

### Study areas

#### Nietoperek site (MRU)

Natura 2000 site PLH080003 “Nietoperek” lies in Lubuskie voivodeship in western Poland (central point: E 15.480600, N 52.394400). It spreads over 7377.37 ha above a 32-km underground system of tunnels (“Międzyrzecki Rejon Umocniony”, MRU) and on ground fortifications built in the 30 s by Germany, as a part of a larger defensive front “Ostwall” or “Festungsfront im Oder-Warthe Bogen”^3,28^. Currently abandoned by the military it is a critical hibernation site for bats in central Europe. With a record count exceeding 39,000 individuals of 12 species including over 600 western barbastelles, it is among the EU's top 10 largest hibernation sites^3,28^. The landscape above the tunnels mainly consists of young monocultures of Scots pine *Pinus sylvestris* (Linné, 1753) mixed with isolated patches of riparian woodlands with ash *Fraxinus* sp. and alder *Alnus* sp. Remaining mostly in depressions and along shorelines of lakes and river areas with restricted access for heavy timber harvesting machinery. This habitat covers approximately half of the protected area (46.19%, ca. 3400 ha). The remaining half is a rural landscape of fields, shrubs and woodlots with willows within cropland. The vegetation period in this part of Poland lasts for approximately 230 days^29^.

#### Schrony Brzeskiego Rejonu Umocnionego site (BRU)

Natura 2000 site PLH200014 “Schrony Brzeskiego Rejonu Umocnionego” (“Shelters of the Brest Fortified Line”, BRU) located in Podlaskie voivodeship, eastern Poland (central point: E 22.994400, N 52.392100) cover ca. 126 ha. In this area, there are 15 iron-concrete bunkers approximately 2 km west of the village of Anusin and 4 km to the east near Moszczona Królewska. The bunkers were built in the 1940s by the Soviet Union as part of a defensive line against the expected invasion of Nazi Germany (the so-called Molotov Line). They are a hibernation site for a winter population of *Barbastella barbastellus* (approximately 400 individuals) and several other bat species. It is an essential place for bat hibernation in this part of the country. The characteristic features of the landscape are the elevations of the postglacial moraines and river bends. Local forest habitats are sandy and consist mainly of Scots pine. Discontinuous pine forests surround the bunkers, and there are also a few fragments of riparian vegetation, including willows. There are small villages and agricultural areas in the vicinity. The vegetation period in this part of Poland lasts for approximately 215 days^29^.

### Bioacoustics

We used a combination of four Song Meter SM4 Wildlife Audio Recorders (Wildlife Acoustics, USA) and six Audiomoths (Open Acoustic Devices, UK). The SM4s were deployed in two pairs in the “Nietoperek” site for 25 nights beginning on March 10th 2022. The Audiomoths were deployed in the same paired design in “Schrony Brzeskiego Rejonu Umocnionego” for 6–13 nights starting March 18th 2022. Detectors were placed after the first blossoming willows were observed in each study area. A pair consisted of one detector mounted on a willow tree with a microphone pointing upwards, and the other one installed similarly on a non-willow tree, i.e. non-early blossoming species, at least 200 m^30^ from and no more than 300 m away from the paired device to ensure the probability of recording independent data while maintaining similar geographic features. Pairs within a single study area were at least 500 m apart and no more than 3 km away (Table 1). The landscape in both study areas was best described as agricultural land with scrub bordering managed forest. The distance between the study areas was ca. 550 km. The geographic distance between both sites resulted in a phenological shift between them. All detectors were programmed to record from dusk till dawn, adjusted to their geographical location. The measure of activity was the number of bat passes. The term "bat pass" means a single flight of a bat understood as one well-defined series of echolocation signals. We take two or more single echolocation pulses as a series of signals. The series are separated by a minimum of 1 s between one and the next^31^. Recordings were manually identified to species, where possible and otherwise to genus, in BatSound 4.2 software (Pettersson. Elektronik AB, Sweden).

**Table 1:** Exact locations of detector deployment.

Site	No. of paired nights	Willow		Control	
		Latitude	Longitude	Latitude	Longitude
BRU	6	52.225504	22.565710	52.225877	22.564399
BRU	11	52.225644	22.562802	52.230556	22.562561
BRU	13	52.223872	22.561699	52.224653	22.560879
MRU	25	52.356946	15.453762	52.355784	15.453696
MRU	25	52.327864	15.438774	52.328997	15.438510

*BRU* Schrony Brzeskiego Rejonu Umocnionego site, *MRU* Międzyrzecki Rejon Umocniony site.

### Statistical analysis

We used a linear mixed effects modelling approach to determine (i) whether bats selected willow tree species sites over non-willow tree species sites, and (ii) explore the importance of willow tree species sites for bats over survey nights. We first built a model that included western barbastelle calls as the response variable, and then built a separate model for ‘other’ recorded bat calls as the response variable, using the nlme R package^32^. The data was not transformed prior to model building and normality was not tested for due to the small sample size. The distribution of the residuals of each fitted model were visually checked for normality using a Q-Q plot; residuals did not deviate from normal. Homogeneity of variance was assessed using residual plots. The spread of residuals was even. Detector position (willow tree species site or non-willow tree species site) and survey night were included as fixed effects, and paired site included as a random effect. Models were fitted using Restricted Maximum Likelihood (REML). Model terms were tested for importance using ANOVA. All statistical analyses were performed in R 4.1.2 and RStudio 1.0.143^33^.

### Research ethics

Our research relied on non-invasive passive recording devices which do not require specific institutional animal care approval.

## Results

**Figure 1:**
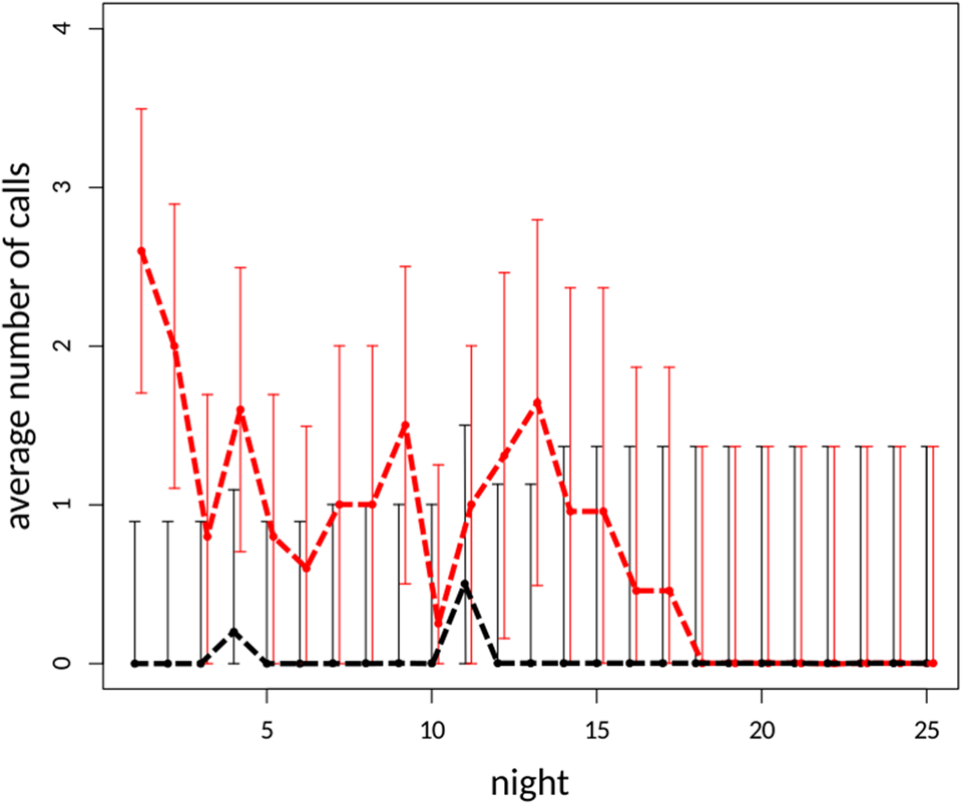
Estimated mean averages of the western barbastelle (*Barbastella barbastellus*) calls in willow tree species sites (red) and non-willow tree species sites (black) over time, derived from linear mixed effects regression models. Dashed lines of corresponding colour represent trend lines for given categories. Error bars indicate 95% confidence intervals.

**Figure 2:**
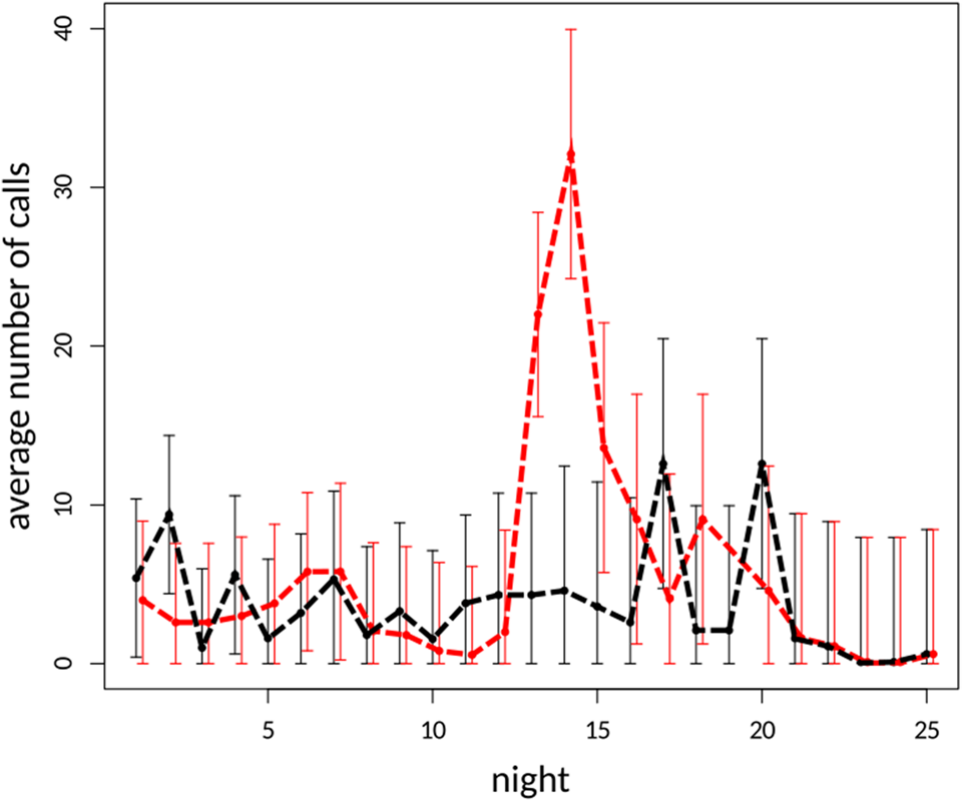
Estimated mean averages of ‘other’ bat calls in willow tree species sites (red) and non-willow tree species sites (black) over time, derived from linear mixed effects regression models. Dashed lines of corresponding colour represent trend lines for given categories. Error bars indicate 95% confidence intervals.

A total of 160 nights of bat recordings were made consisting of 80 nights at the combined five paired sites. Of these 80 night recording periods there were nine nights in which no bats were recorded in either the willow or control treatments. For the remaining 71 nights bat echolocation calls were recorded. When assessing the difference in the western barbastelle calls between willow and non-willow tree species sites our model identified a significant difference. The number of recorded calls at willow sites was significantly greater when compared to the number of recorded calls at non-willow sites (F(1,152) = 40.54, p < 0.001) (Fig. 1, Table 2). No significant difference was identified between willow and non-willow tree species sites when ‘other’ bat calls were analysed (F(1,152) = 2.72, p = 0.1) (Fig. 2). When assessing the change in the western barbastelle occurrence at willow tree species sites over survey nights we found a significant decrease in recorded western barbastelle calls from first survey night (F(1,152) = 13.55, p < 0.001). The number of western barbastelle calls were relatively high at the start of the survey period with incremental nightly decreases (Fig. 1). In contrast, when assessing the change in ‘other’ bat calls at willow sites over time we found borderline significance and an increase in recorded calls over time (F(1,152) = 3.90, p = 0.0501) (Fig. 2).

**Table 2:** Results of the linear mixed effects models used to test the effect of position (willow or non-willow tree), night and position over survey nights.

Independent	lme					ANOVA	
	Predictor	Estimate	SE	t-value	p	f-value	p
*B. barbastellus*	Intercept	0.064	0.202	0.319	0.750	19.506	
	Willow	1.695	0.256	6.612	0.000	40.543	< 0.001
	Night	− 0.004	0.015	− 0.296	0.768	14.502	0.000
	Willow:night	− 0.076	0.021	− 3.681	0.000	13.553	0.000
Other	Intercept	4.821	1.808	2.666	0.009	40.354	
	Willow	− 1.803	2.521	− 0.715	0.476	2.722	0.101
	Night	− 0.101	0.144	− 0.700	0.485	0.920	0.339
	Willow:night	0.399	0.202	1.975	0.050	3.901	0.050

Results of model terms tested for importance using ANOVA are also presented.

## Discussion

The relationship we found between an early-flowering tree species and a specialized bat species (the moth foraging specialist western barbastelle) is an example of a chain of dependencies formed between different groups of organisms. In early spring, *Salix* sp. is a key genus on which many species depend, primarily as a food resource and secondly as it attracts prey for insect-eating species such as birds and bats^20,34^. It can therefore be assumed that the increased activity of barbastelle around flowering willows is associated with the presence of potential food. The willow moths, like *Orthosia* (Noctuidae)*,* are an established component of the barbastelles diet and have been found in their guano collected in early April^13^. *Orthosia* moths hibernate in adult form, ready for activity and end their dormant period early in spring. Thus, they appear early on the flowers of *Salix* sp., on which they feed. The western barbastelle is a species whose hibernation period ends earlier than that of other Palearctic bats^5^ and is a moth specialist adapted to capture Noctuidae^14^.

*Salix caprea*, like other Palearctic willow species—with which it often hybridizes—is pollinated by wind and diurnal and nocturnal insects^20^. The diurnal insects are mainly *Hymenoptera* and *Diptera* species, and the nocturnal insects are mostly Noctuidae moths. The latter, especially the genus *Orthosia* is specialised in feeding on *Salix* sp., and is therefore, also an important pollinator for the tree^20,24,25^.

The relationship between the willow and the bat is probably one-sided because bats benefit from feeding on moths attracted by willows. At the same time, this phenomenon is likely indifferent to the tree's survival. The loss of pollinating insects is negligible, considering the high prevalence of *Salix* trees and shrubs and the relatively low density of western barbastelles in the landscape. In this case, it would be a simple relationship where flowers provide food for a specialized insect species, which in turn is early spring prey for the western barbastelle (commensalism).

The western barbastelle leaves winter roosts early. Although the available literature is very sparse^5^, this species likely ends its hibernation in March, generally. Moreover, it is already known that during hibernation, bats break their torpor, drink water, and also forage, replenishing their energy reserves^35^. Therefore, it is unclear whether the animals we recorded were feeding over *Salix* sp. trees during the final stage of hibernation or immediately afterwards. However, food availability is considered one of the major factors influencing the spatial–temporal patterns of bat activity^36^. We found that the period of occurrence of barbastelles in the vicinity of flowering willows was relatively short. After 1 week, their activity decreased, and the barbastelles mostly disappeared (Fig. 1). Therefore, we conclude that barbastelles at that time used temporary roosts, which may be the neighbouring hibernation sites. The areas surrounding both study plots were covered by a forest composed mainly of young (x < 50 years) pines (https://www.bdl.lasy.gov.pl/portal/mapy). Such a habitat does not offer good roosting opportunity for the barbastelle, which in summer uses cracks in broken trunks or space behind protruding bark^37–39^ and in some cases also buildings, especially wooden ones^40–42^. Regardless, if we take the results of our research as evidence, spring foraging by willows will provide a temporary but critical supply of prey—allowing fast regeneration just after hibernation.

It is recognized that diversity in a rural landscape is an integral part of biological diversity, also in an area of Europe where agricultural activity has a long history and the vast majority has been permanently transformed. For this reason, efforts are made to protect and increase diversity for economic and biological reasons. The list of agricultural landscape elements important for maintaining diversity is still expanding^43–47^, and bats are one of the subjects of interest^48–53^, both as an element of the ecosystem and as a factor regulating the number of insects^54–60^. Willows are a frequent element of Europe's rural landscape (which includes agricultural areas often mixed with tree stands and fragmented woodlands) appearing both as large trees and as shrubs, especially in meadows and wetlands. In many regions with a temperate climate, they are part of the traditional landscape of pastures and meadows, which is an important factor in the conservation of bats^61^. Our research shows that willows can play an important role in the ecosystem for some species of bats. This is evidence for the protection of moth specialist bats such as western barbastelles and possibly long eared bats *Plecotus* sp., as willow tree stands growing in the area where hibernation sites of these species are located should be treated as an important element for local bat populations, and thus should be protected against removal or degradation. We also propose that complementary planting of willow in the vicinity of bat wintering grounds will increase the potential prey availability at a critical time of the year.

## Data Availability

The datasets generated during and/or analysed during the current study are available from the corresponding authors on reasonable request.
